# Recurrent growth factor starvation promotes drug resistance in human leukaemic cells

**DOI:** 10.1038/sj.bjc.6600036

**Published:** 2002-01-21

**Authors:** K Saeki, E Okuma, A Yuo

**Affiliations:** Department of Hematology, Research Institute, International Medical Center of Japan, 1-21-1 Toyama, Shinjuku-ku, Tokyo 162-8655, Japan

**Keywords:** leukaemia, MO7e, GM-CSF, multi-drug resistance

## Abstract

Multi-drug resistance can be induced by various environmental stresses including an exposure to chemical drugs and X-ray irradiation. In addition, hypo-nutritive conditions are known to promote multi-drug resistance in solid tumours. To understand the importance of nutritive conditions in the development of drug resistance in non-solid tumours and to know whether a transient malnutrition could induce a permanent reduction in drug sensitivity, leukaemic cells were transiently cultured under growth factor-starved conditions. Granulocyte-macrophage colony-stimulating factor-dependent human leukaemic MO7e cells were cultured in the absence of granulocyte-macrophage colon-stimulating factor for 2 weeks, during which the majority of the cells died, and the minor viable cells were expanded in the presence of granulocyte-macrophage colon-stimulating factor for following 1 week. This procedure was repeated three times, and the surviving cells were cloned by limiting dilution. These clones underwent G1 arrest in the absence of granulocyte-macrophage colon-stimulating factor, while parental cells underwent apoptosis. Interestingly, activities of the downstream targets of granulocyte-macrophage colon-stimulating factor receptor were regulated in a granulocyte-macrophage colon-stimulating factor-independent manner, indicating that the ligand-independent activation of granulocyte-macrophage colon-stimulating factor receptor had not taken place. Moreover, the 4–7-fold increases in IC_50_ for etoposide and the 2–6-fold increase in IC_90_ for doxorubicin was observed. Furthermore, Bcl-2 protein expression was significantly up-regulated in the clones while no significant changes in Bax, Bcl-_xL_, P-glycoprotein and Hsp70 protein expression and no consistent changes in p53 expression were detected. We propose that recurrent growth factor starvation, which may occur *in vivo* when stromal function is damaged after intensive chemotherapy or bone marrow occupation by malignant cells, causes selection of drug resistant leukaemia cells that will expand when the growth factor supply recovers.

*British Journal of Cancer* (2002) **86**, 292–300. DOI: 10.1038/sj/bjc/6600036 www.bjcancer.com

© 2002 The Cancer Research Campaign

## 

Multi-drug resistance is one of the most serious problems in the treatment of malignant tumours. Although studies have revealed its complicated mechanisms including enhancement in drug export by ATP-binding cassette transporter proteins (reviewed by Ross, 2000), activated drug detoxification by glutathione S-transferase (reviewed by Zhang *et al*, 1998), reduction in cell death commitment by loss or mutation of p53 (Aas *et al*, 1996 and Ju *et al*, 1998), topoisomerase II down-regulation (Shen *et al*, 1989; Yun *et al*, 1995) and apoptosis inhibition by over-expression of Bcl-2 family proteins (reviewed by Reed, 1997), triggering events that induce multi-drug resistance are not completely understood. It has been shown that the hypo-nutritive conditions promote a transient multi-drug resistance in solid tumours. The local hypoglycaemia or hypoxia induces multi-drug resistance although nutritive improvement instantaneously rescues the drug resistance (Hughes *et al*, 1989; Lee, 1992; Shen *et al*, 1987; Tomida *et al*, 1996). Indeed, hypo-nutritive conditions occur *in vivo* in solid tumours with an enlargement of tumour size and subsequent blood supply loss (Hockel *et al*, 1996). By contrast, leukaemic cells are seldom put under such conditions because they grow in a disseminate manner in blood flow or bone marrow, where nutrients are abundant, and also leukaemia demonstrates neoangiogenesis. On the other hand, growth-factor-deficient conditions may occur to leukaemic cells *in vivo* when stromal functions are severely damaged after intensive chemotherapy or bone marrow occupation by malignant cells (Ben-Ishay *et al*, 1990).

In the present study, we studied the effects of a transient growth factor deficiency on drug sensitivities using Granulocyte-macrophage colony-stimulating factor (GM-CSF)-dependent human leukaemic MO7e line, which was established from magakaryocytic leukaemia patient as the cells whose growth completely depends on GM-CSF or interleukin-3 (Avanzi *et al*, 1990). We show that a recurrent growth factor starvation results in a permanent reduction in drug sensitivity. The molecular mechanism and the clinical relevance of this phenomenon will be discussed.

## MATERIALS AND METHODS

### Cells, the cytokine and drugs

Human leukaemic MO7e cells were maintained in RPMI1640 (Life Technologies, Inc., Grand Island, NY, USA) supplemented with 10% heat inactivated foetal calf serum (FCS) and also 10 ng ml^−1^ of recombinant human GM-CSF, which was provided by Kirin Brewery Company, Ltd. (Tokyo, Japan). Human leukaemic K052 cells were provided by Health Science Research Resources Bank (Osaka, Japan) and maintained in α-MEM (Life Technologies, Inc., Grand Island, NY, USA) supplemented with 10% heat inactivated foetal calf serum. Doxorubicin, etoposide and actinomycin D were purchased from Sigma Chemical Co. (St. Louis, MO, USA). In short drug exposure experiments, relatively high doses of drugs were used. The 5 μg ml^−1^ of etoposide or 1 μg ml^−1^ of doxyrubicin, both of which are used clinically (Aydiner *et al*, 2000, Battista *et al*, 1991), and 1 μg ml^−1^ of actinomycin D, which is commonly used in *in vitro* apoptosis-inducing experiments (Cotter *et al*, 1992), were added to the cells, and the cell viability was tested after incubation for 6 h or 14 h.

### Preparation of the growth factor withdrawal-resistant MO7e sublines

**Figure 1:**
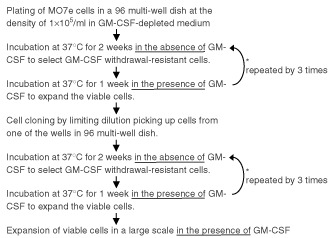
The flow diagram of the GM-CSF withdrawal-resistant cell cloning procedure.

The flow chart of the procedure (Figure 1

). First, MO7e were seeded in a 96-multiwell plate with aliquot volume of 100 μl at the density of 1×10^5^ ml^−1^ in the absence of GM-CSF and then cultured at 37°C for 2 weeks. Compatible to the previous report that the growth of MO7e completely depends on GM-CSF (Avanzi *et al*, 1990), the majority of MO7e died within 4 days after GM-CSF deprivation and we could hardly detect the viable cells by direct microscopic observation at day 14. Then we added GM-CSF to each well to expand the viable cells if they existed. After 1 week's expansion, we found that several wells contained viable cells. These cells were cultured again in the absence of GM-CSF as the 2nd selection. After 2 weeks of incubation, GM-CSF was added again to expand viable cells. This procedure was repeated three times. After the final expansion, we found that 12 out of 96 wells contained viable cells. As these cells formed multi-focus in the wells, we performed the clonal isolation by limiting dilution in a 96-multiwell plate picking up cells from the well that contained the largest number of viable cells. After seeding of the cells, the three cycles of GM-CSF withdrawal/re-addition procedure were performed as before. After the final expansion, eight clones were obtained. These sublines were maintained in the presence of GM-CSF as well as parental cells.

### Reverse transcription–polymerase chain reaction (RT–PCR)

Messenger RNA was extracted from 8×10^7^ cells by Fast Track 2.0 Kit (Invitrogen Co. Carsland, CA, USA) and suspended in 50 μl of 0.1% diethyl pyrocarbonate-containing water. The 1 μl of mRNA solution was used for reverse transcriptase reaction with AMV reverse transcriptase XL (Takara Shuzo Co., Ltd. Shiga, Japan) and the product was suspended in 20 μl of DEPC water. PCR was carried out by Bio Medical Laboratories Co. Ltd. (Tokyo, Japan) using DNA Thermal Cycler PJ2000 (The Perkin-Elmer Co. Foster City, CA, USA) with 16 pmol ml^−1^ of MDR1 sense primer (5′-AAGCTTAGTACCAAAGAGGCTCTG-3′), 16 pmol ml^−1^ of MDR1 antisense primer (5′-GGCTAGAAACAATAGTGAAAACAA-3′), 4 pmol ml^−1^ of β2-microglobulin sense primer (5′-GTGGAGCATTCAGACTTGTC-3′) and 4 pmol ml^−1^ of β2-microglobulin antisense primer (5′-CTGCTTACATGTCTCGATCC-3′) The PCR program was 94°C; 30 s, 55°C; 1 min, 72°C; 1 min for 30 cycles. The product was separated by agarose gel electrophoresis and the DNA (the product sizes: MDR1; 243 bp, β2-microglobulin; 160 bp) was visualized by ethidium bromide. For molecular marker, smart ladder (0.2 k–10 kbp) (WAKO Pure Chemical Industries, Osaka, Japan).

### Evaluation of apoptosis

DNA fragmentation assay was performed by extracting the low molecular weight DNA and visualizing the internucleosomal DNA breakdown as previously described (Saeki *et al*, 1997). The magnitude of apoptosis was quantified by calculating percentage of hypodiploid cell population in DNA content analysis by using fluorescence-activated cell sorter (FACS) as described previously (Okuma *et al*, 2000). Morphological study was performed by direct microscopic observation with phase contrast examination (Olympus Optical Co. Ltd., Tokyo, Japan).

### Assessment of clonogenisity

Cells were seeded at the density of 1×10^2^ ml^−1^ in 24-well multi-well dish and cultured in CO_2_ gas incubator. After 2 weeks, numbers of the colonies that consist of more than 50 cells were counted.

### Determination of 50% inhibitory concentrations (IC_50_) and 90% inhibitory concentrations (IC_90_) by 3 days WST assay

Cells were cultured at 3×10^5^ ml^−1^ in 24-multiwell dish in the presence of GM-CSF and various doses of drugs (1, 2, 4, 8, 10, 20, 40, 80 ng ml^−1^ of doxorubicin; 2.5, 5, 10, 20, 40 ng ml^−1^ of etoposide; 5, 10, 20, 40, 80 pg ml^−1^ of actinomycin D). After 72 h, the viability of the cells was assessed by 2-(2-methoxy-4-nitrophenyl) - 3 - (4- nitrophenyl)-5-(2,4-disulfophenyl)-2H-tetrazolium (WST)-8-reducing activity using Cell Counting Kit-8 (WAKO Pure Chemical Industries) according to the manufacturer's protocol. Briefly, aliquot volume of the cells were translocated into 96-multiwell dish and incubated with WST solution. After 2 h, the absorbance at 490 nm (A490) and at 595 nm (A595) was examined. Then the percentages of ΔA values (A490–A595) of the drug-treated cells to those of non-treated cells were calculated. These values were plotted on the 2-dimensioned graph with horizontal axis for drug doses and vertical axis for relative ΔA values. The IC_50_ and IC_90_ were determined as the dose that gave the 50 and 90% ΔA value, respectively. The results from independent triplicate experiments were used for the determination of IC_50_.

### Immunostaining of p-glycoprotein

Immunostaining of p-glycoprotein was performed as described previously (Saeki *et al*, 2000) using cells fixed on slide glasses by cytospin apparatus (Cytospin2, SHANDON, Pittsburgh, PA, USA), which were further fixed with acetone : methanol solution (1 : 3). The fixed cells were incubated with 50 μl of PBS containing anti-p-glycoprotein monoclonal antibody (1 : 100 dilution) (DAKO A/S, Denmark) and FCS (1 : 200 dilution) at 37°C for 60 min, washed with 10 ml of PBS for 5 min by three times and further incubated with 50 μl of PBS containing FITC-conjugated gout anti-mouse IgG (1 : 50 dilution) (CHEMICON International, Inc., Temecula, CA, USA) and FCS (1 : 100 dilution) at 37°C for 60 min. After washing them with PBS by five times, cells were observed under fluorescent microscopy (Olympus Optical Co. Ltd., Tokyo, Japan). The stained cells also observed by light microscopy with Normarsky differentiated interference contrast (Olympus). For a positive control, human leukaemic K052 cells (Health Science Research Resources Bank, Osaka, Japan) were used.

### GM-CSF stimulation and Western blotting

After 20 h incubation in the absence of GM-CSF, MO7e cells stimulated with GM-CSF (10 ng ml^−1^). After 5 min incubation at 37°C, cells were collected, lysed by 1× Laemmli's sample buffer and boiled. Aliquot (i.e. 5×10^4^ cells) was used for Western blotting. First antibody reaction was performed using anti-phosphorylation-specific Stat 5 monoclonal antibody (New England Biolabs, Inc., Beverly, MA, USA), phosphorylation-specific Akt polyclonal antibody (New England Biolabs, Inc.) and phosphorylation-specific monoclonal extracellular signal-regulated kinase (ERK) antibody (New England Biolabs, Inc.). Anti-bcl-2 monoclonal antibody (Pharmingen, San Diego, CA, USA), anti-HSP70 monoclonal antibody (Santa Cruz Biotechnology Inc., Santa Cruz, CA, USA), anti-p53 monoclonal antibody (Santa Cruz Biotechnology Inc.) and anti-p21 monoclonal antibody (Pharmingen, San Diego, CA, USA) and anti-topoisomerase II α polyclonal antibody (Santa Cruz Biotechnology Inc.) were also used in other experiments. Second antibody reaction and the final detection procedure were performed as previously described (Saeki *et al*, 1997).

### Examination of the surface expression of CD61 by fluorescence-activated cell sorting (FACS)

The 5×10^5^ cells were collected, washed twice by PBS containing 10% foetal calf serum and then incubated with 10 μl of FITC-conjugated anti CD61 antibody (DAKO A/S, Denmark) or 10 μl of control FITC-conjugated mouse IgG1 (Beckton Dickinson, Mountain View, CA, USA) at 4°C for 30 min. Then, cells were washed with 10% foetal calf serum-containing PBS for four times, and the CD61 expression was analyzed by FACScalibur (Beckton Dickinson).

### Karyotype analysis

Karyotype analysis was performed by Bio Medical Laboratories Co. Ltd. (Tokyo, Japan) by the conventional method (Benn and Perle, 1992). Chromosomes were stained with Giemsa solution and examined by a microscope. Twenty metaphase spreads were examined in each sample.

### Fluorescence *in situ* hybridization (FISH) analysis

Cells were fixed, denatured, hybridized with specific chromosomal probes (WCP 17 SpectrumOrange and WCP 22 SpectrumGreen) according to the manufacturer's protocol (Vysis, Inc., IL, USA) and photographed through fluorescent microscopy by Bio Medical Laboratories Co. Ltd. (Tokyo, Japan).

## RESULTS

### Acquisition of growth-factor-withdrawal-resistant clones

To know whether malnutritive conditions, including growth factor deficiency, would possibly promote drug resistance in leukaemia, we tried to select clones that had adapted to growth factor deprivation using GM-CSF-dependent human megakaryocytic leukaemic MO7e cells as described in Materials and methods. We could actually obtain eight clones after the final selection. We randomly selected three clones (cl-1, cl-2, and cl-3) and maintained them in the presence of GM-CSF. Chromosomal analysis confirmed that they were derivatives of MO7e and not the contamination of other cell lines (Table 1

**Table 1:**
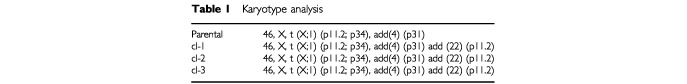
Karyotype analysis

). Using these three clones, we determined the resistance to GM-CSF withdrawal. All these clones survived a significantly longer time than the parental cells in the absence of GM-CSF (Figure 2A

**Figure 2:**
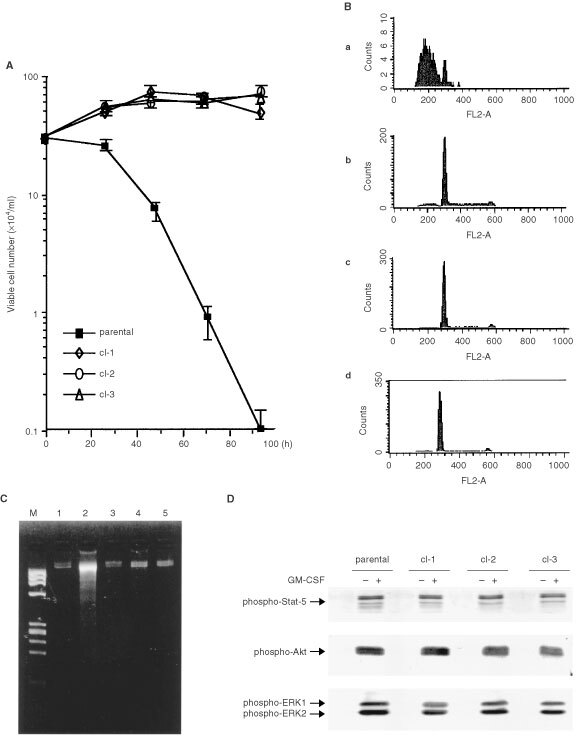
MO7e sublines with elongated life spans in the absence of GM-CSF but with normal intracellular signalling after GM-CSF receptor. (**A**)–(**C**) Parental MO7e cells and the three sublines (cl-1, cl-2 and cl-3) were cultured in the absence of GM-CSF at the density of 3×10^5^ ml^−1^ in a 24 multi-well plate. (**A**) Viable cell number was counted in time course. (**B**) DNA content analysis was performed at day 4 (a; parental cells, b; cl-1, c; cl-2, d; cl-3). (**C**) DNA fragmentation assay was performed at day 1 (M indicates the molecular marker. Lane 1; parental cells cultured with GM-CSF, lane 2; parental cells cultured without GM-CSF, lane 3; cl-1, lane 4; cl-2, lane 5; cl-3 cultured without GM-CSF. (**D**) After 20 h starvation of GM-CSF, parental cells and the sublines were stimulated by water (indicated as −) or by 10 ng ml^−1^ of GM-CSF (indicated as +) for 5 min. Lysate was prepared from aliquot number of cells and Western blotting was performed using anti-phosphorylated Stat-5 (Tyr 694) antibody (upper), anti-phosphorylated Akt (Ser 473) antibody (middle) and anti-phosphorylated ERK (Thr 202/Tyr 204) antibody (lower).

). They underwent G1 arrest at day 4 after GM-CSF starvation (Figure 2B), while parental cells underwent apoptosis as early as day 1 as determined by DNA fragmentation assay (Figure 2C). To exclude the possibility that an enhanced survival in the absence of GM-CSF comes from a ligand-independent activation of GM-CSF receptor, activities of its downstream target molecules were examined. As shown in Figure 2D, the phosphorylation of transducer and activator of transcription 5 (Stat 5), Akt and extracellular signal-regulated kinase (ERK) were all regulated in a GM-CSF-dependent manner, indicating that GM-CSF withdrawal resistance was not due to the ligand-independent activation of GM-CSF receptor.

Thus, the growth factor withdrawal-resistant MO7e clones were obtained after repetitive growth factor starvation.

### Growth-factor-withdrawal-resistant clones showed drug resistance

**Figure 3:**
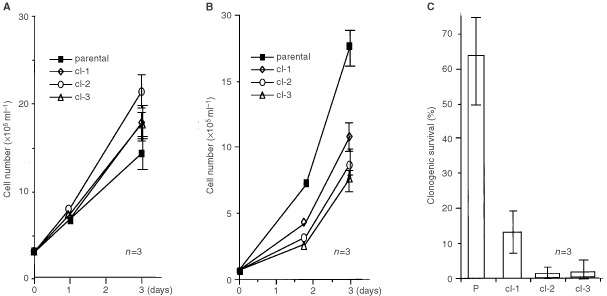
The growth rate of MO7e sublines depends on their cell density. Parental cells (▪) and sublines (cl-1; ⋄, cl-2; open circle, cl-3; Δ) were cultured in the presence of GM-CSF at the density of 3×10^5^ ml^−1^ (**A**), 7×10^4^ ml^−1^ (**B**) and 1×10^2^ ml^−1^ (**C**) in 24 multi-well dish. Viable cell number was counted in time course (**A** and **B**) or number of the colonies was counted at day 14 (**C**). Results of three independent experiments were shown.

We next compared the sensitivities to chemical drugs in the presence of GM-CSF among parental cells and the growth-factor-withdrawal-resistant clones. Because cell cycling speed often affects the drug sensitivities, we checked the growth rate of parental cells and sublines in the presence of GM-CSF. When cells were seeded at a relatively high density of 3×10^5^ ml^−1^, growth speed of the sublines was not at all slower, or even faster, than that of the parental line (Figure 3A

). On the other hand, the growth rate of sublines decreased when seeded at a relatively low density of 7×10^4^ ml^−1^ (Figure 3B). Moreover, the growth of sublines remarkably reduced and, as a result, the clonogenic activity became considerably lower when seeded at a lowest density of 1×10^2^ ml^−1^ (Figure 3C). For this reason, the following experiments were performed at the density of 3×10^5^ ml^−1^, where growth rate of parental cells and sublines did not significantly differ.

**Figure 4:**
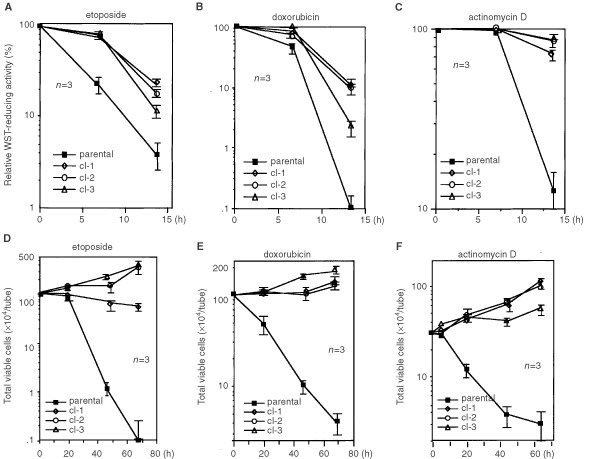
A short-term WST assay and a transient drug exposure assay. (**A**)–(**C**) Parental cells and sublines were cultured at the density of 3×10^5^ ml^−1^ in the presence of etoposide (5 μg ml^−1^) (**A**), doxorubicin (1 μg ml^−1^) (**B**) or actinomycin-D (1 μg ml^−1^) (**C**), and WST assay was performed as indicated time points. Horizontal axis indicates the percentages of WST-reducing activities of drug-treated samples to those of drug-untreated one in each cell line. (**D**)–(**E**) The 3×10^5^ ml^−1^ of parental cells and the sublines were transiently treated with etoposide (2.5 μg ml^−1^) for 30 min (**D**), doxorubicin (0.5 μg ml^−1^) for 30 min (**E**) or actinomycin D (1 μg ml^−1^) for 6 h (**F**), and then cells were washed. After 24 h incubation, cells were diluted. After incubation for indicated time, the number of total viable cells was counted. Results of the three independent experiments were shown. (▪: parental cells,⋄: cl-1, ○: cl-2, Δ: cl-3).

**Figure 5:**
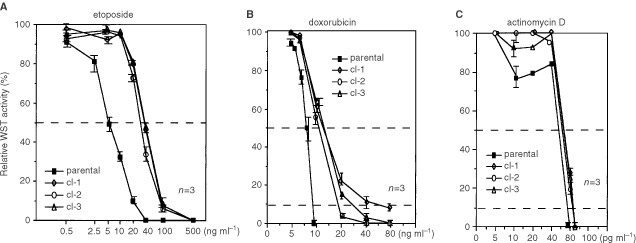
The 3 days WST assay for the determination of IC_50_ and IC_90_. The 3×10^5^ ml^−1^ of parental cells (▪) and sublines (cl-1; ⋄, cl-2; ○, cl-3; Δ) were treated with etoposide (**A**), doxorubicin (**B**) and actinomycin D at indicated doses, and WST assay was performed at day 3. Horizontal axis indicates the percentages of WST-reducing activities of drug-treated samples to the untreated ones in each line. The results from the three independent experiments were shown.

First we performed a short-term WST assay using relatively high doses of drugs (i.e. 5 μg ml^−1^ of etoposide, 1 μg ml^−1^ of doxorubicin or 1 μg ml^−1^ of actinomycin D). The cell viability was examined after an incubation for 6 h or 14 h by WST asssay. We found that sublines showed higher viability than parental cells at any time point (Figure 4A,B and C

). To check the cell death commitment time in each clone, drugs were transiently added. Practically, cells were exposed to 2.5 μg ml^−1^ of etoposide for 30 min, 0.5 μg ml^−1^ of doxorubicin for 30 min or 1 μg ml^−1^ of actinomycin D for 6 h, cell growth was chased after washing out the drugs. We found that the growth of the sublines recovered while parental cells did not grow but died (Figure 4D,E and F). To evaluate the magnitude of drug resistance, the 3 days WST assay was performed and IC_50_ value was determined. Etoposide, doxorubicin or actinomycin D was added to the cells at indicated doses with dilution factor of 2, and the relative WST-reducing activities of drug-treated samples to those of non-treated ones were assessed at day 3. As shown in Figure 5A

, viability curves of sublines for etoposide were apparently shifted to the right, and IC_50_ values were increased by 4–7-fold (Table 2

**Table 2:**
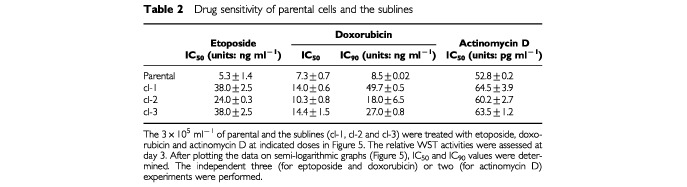
Drug sensitivity of parental cells and the sublines

). Although no significant change in IC_50_ values for doxorubicin was detected (Table 2), we found slopes in the viability curves of the sublines at higher doses (Figure 5B). Actually, IC_90_ values were increased by 2–6-fold in the sublines (Table 2). For actinomycin D, there was no increment in either IC_50_ or IC_90_ values (Figure 5C).

Thus, GM-CSF-withdrawal-resistant sublines showed the short-term resistant to etoposide, doxorubicin and actinomycin D with delays in cell death commitment time and the long-term resistant to etoposide and doxorubicin.

### Expressions of drug-resistance-associated genes and a differentiation marker

**Figure 6:**
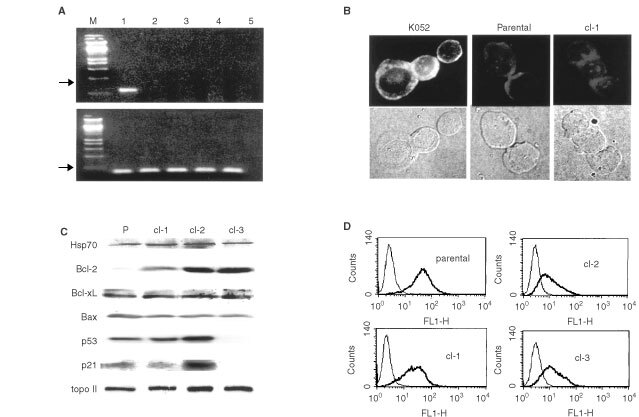
The expressions of drug resistance-related genes and the differentiation marker. (**A**) The mRNA was extracted from mdr1-positive K052 cells (lane 1), parental cells (lane 2), cl-1 (lane 3), cl-2 (lane 4) and cl-3 (lane 5), and the expression of mdr1 message (upper) or β2 microglobulin (lower) was examined by RT–PCR. M indicates the DNA molecular marker. (**B**) The mdr1-positive K052 (left) parental cells (middle) cl-1 (right) were fixed, and p-glycoprotein expression was examined by immunostaining (upper). Lower panels indicate the photographs by light microscopy with Normarsky differentiated interference contrast. (**C**) Lysate of equivalent numbers of parental cells and the sublines were prepared and Western blotting was performed using antibodies against Hsp70, Bcl-2, Bcl-xL, Bax, p53, p21 and topoisomerase IIα. (**D**) The surface expression of CD61 was examined by FACS. The thin lines indicate the staining with control FITC-conjugated mouse IgG and the bold lines indicate the staining with anti-CD61 antibody.

To understand the molecular mechanism of drug resistance in the clones, expressions of drug resistance-related genes were examined. The expression of mdr1, an important gene for drug export, was examined, but it was neither detected in sublines nor parental cells by RT–PCR (Figure 6A

) or immunocytochemistry (Figure 6B). Then we tested the expression of hsp 70, a regulator of heat shock and chemical drug. The protein expression of Hsp 70 did not differ among parental cells and sublines (Figure 6C). We also checked the expression of bcl-2 family proteins, regulators of drug-induced apoptosis, and found a mild up-regulation in cl-1 and remarkable up-regulation in cl-2 and cl-3 in Bcl-2 protein expression without a significant alteration in other bcl-2 family protein expressions (Figure 6C). We also examined the expression of p53, which reportedly renders cells drug-sensitive (Aas *et al*, 1996 and Ju *et al*, 1998), and a complicated result was obtained; cl-1 with a similar p53 expression as in parental cells; cl-2 with a significantly up-regulated p53 expression; cl-3 with almost loss of p53 expression (Figure 6C). To know the activity of p53, we examined the expression levels of p21, a downstream target of p53. We found that the protein expression level of p21 precisely paralleled with that of p53 (Figure 6C), suggesting that p53 had wild type transcriptional activity in each line although p53 expression did not show correlation with drug resistance. Moreover, we could not detect significant differences in topoisomerase II α expression among parental and sublines (Figure 6C). We further checked the differentiation states of parental cells and sublines because drug sensitivity can reportedly be affected by differentiation (Costello *et al*, 2000; Ohashi *et al*, 2000). The surface expression of megakaryocytic differentiation marker of CD61 was examined, and we found a slight decrement in CD61 expression in sublines in reciprocal proportion to the Bcl-2 expression level (Figure 6D).

Thus, an up-regulation of Bcl-2 protein expression and a slight down-regulation of CD61 was a common feature detected in GM-CSF-withdrawal-resistant clones.

### FISH analysis

**Figure 7:**
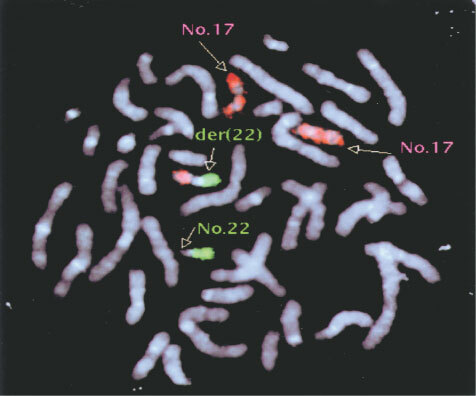
FISH analysis. One of the GM-CSF withdrawal-resistant subline (cl-1) was fixed on the slide glass, and FISH analysis using probes for chromosome 17 and chromosome 22 was performed.

We finally performed FISH analysis to understand the chromosomal translocation of add (22) (p11.2) using one of the clones. As shown in Figure 7

, the translocation turned to be der (22) t (17; 22) (q21; p11.2).

## DISCUSSION

We showed that recurrent growth factor starvation promotes the emergence of drug resistant clones in leukaemic cells.

Malnutritive conditions such as anoxia or aglycaemia reportedly promote multi-drug resistance in solid tumours by inducing glucose-regulated set of stress proteins (Hughes *et al*, 1989; Lee, 1992; Shen *et al*, 1987; Tomida *et al*, 1996) or by proteolytically depleting topoisomerase II, a target of etoposide (Shen *et al*, 1989; Yun *et al*, 1995). In these cases, drug resistance persists only in a transient manner, and an improvement in nutritive conditions immediately restores the drug sensitivity. In our case, however, the drug resistance persists even in the presence of GM-CSF. Moreover, we could not detect significant difference in topoisomerase II α (Figure 6C) and topoisomerase II β (data not shown). Thus, distinct mechanisms are working in the GM-CSF withdrawal-resistant clones. We could not obtain any evidence for activated drug export system because the expression of Mdr1 (Figure 6C) nor MRP1 (data not shown) was not detected in either clone. With regard to p53, we did not observe any correlation between its expression and drug resistance: no changes of p53 in cl-1, up-regulation in cl-2 and reduction in cl-3. Thus, p53 may not be involved in drug resistance in our case. On the other hand, Bcl-2 up-regulation can be a good candidate for the cause of drug resistance. Bcl-2 over-expression is reportedly associated with therapeutic resistance and poor outcome in leukaemic cases in clinical fields (Karakas *et al*, 1998). As we showed, Bcl-2 expression was indeed up-regulated in all subclones although there was a difference in the magnitude of up-regulation among the clones; about two-fold up-regulation in cl-1 and four-fold in cl-2 and cl-3 (data not shown). Because even a slight Bcl-2 up-regulation can render cells resistant to apoptosis (Shimura *et al*, 2000), it seems quite possible that a relative weak Bcl-2 up-regulation in cl-1 promoted drug resistance. A recent report, however, showed that bcl-2 over-expression did not rescue the reproductive cell death following drug treatments *in vitro* (Tannock and Lee, 2001). As we showed, the higher the Bcl-2 expression level is, the lower the clonogenic activity (Figure 3C). We speculate that a high expression of Bcl-2 *per se* might be somewhat toxic for clonogenic survival, and thus bcl-2 over-expression failed to rescue drug-induced reproductive cell death *in vitro*. Why is bcl-2 up-regulation clinically associated with therapeutic resistance? We propose a ‘two hits theory’ in bcl-2-associated therapeutic resistance that a certain second hit has compensated the bcl-2-induced clonogenic reduction. It may be of interest to search the gene that rescues the clonogenic activity in bcl-2-over-expressing cells.

It is known that chromosomal abnormalities are often found in drug-resistant leukaemic cells. Our MO7e sublines had a common chromosomal abnormality of add (22) (p11.2). FISH analysis of cl-1 showed that this rearrangement was der (22) t (17; 22) (q21; 11.2) (Figure 7). At 17q21 is located the retinoic acid receptor α(RARα) gene, whose rearrangement contributes to the development of acute promyelocytic leukaemia. Hybridization analysis, however, revealed that RARα gene was not located at the rearranged locus (K Saeki, unpublished observation), so other genes are responsible for the drug resistance if chromosomal translocation could be involved in it.

What events trigger the acquisition of drug resistance *in vivo*? Environmental stresses such as chemical drugs and X-ray irradiation are reported to induce multi-drug resistance (Licht *et al*, 1991). Our results further propose that the starvation of growth factor can be a trigger for it. After an intensive chemotherapy or even during the natural course of leukaemia progression, local growth factor concentration in bone marrow can be low as a result of stromal dysfunction. Under these conditions, growth factor withdrawal-resistant clones might be selected, which can expand after stromal recovery. Although growth factors can be produced outside the bone marrow, their supply must be too short to support the haematopoietic cell growth in the bone marrow because only an extramedullary haematopoiesis can be observed in cases of severe stromal damage such as myelofibrosis. We suggest that chemotherapeutic protocols with least stromal toxicity can possibly prevent the emergence of multi-drug resistant leukaemic cells.
